# Discovering epigenetic changes in response to tungsten-alloy treatment using next-generation sequencing technologies

**DOI:** 10.1186/1753-6561-6-S6-P13

**Published:** 2012-10-01

**Authors:** Ranjana Verma, Deepak Grover, Zygmunt Galdzicki

**Affiliations:** 1 Department of Anatomy, Physiology and Genetics, School of Medicine, Uniformed Services University of the Health Sciences, 4301 Jones Bridge Road, Bethesda, MD 20814, USA

## Background

Heavy metals used in industrial and household applications can pose harmful health effects. Tungsten alloys (WAs) have been widely used in many engineering, automotive and marine applications. In military operations, WAs containing tungsten (W; 91% w/w), nickel (Ni; 6% w/w) and cobalt (Co; 3% w/w) have been deployed in armor-penetrating munitions as substitutes for depleted uranium. Despite widespread use, a clear understanding of the potential effects of WAs on physiological processes and gene expression is not available. WAs have carcinogenic potential as demonstrated by cancer development in rats with intramuscular implanted WA pellets. This suggests a potential involvement of epigenetic events previously implicated as environmental triggers of cancer. In the present study, we have attempted to unravel WA-mediated alterations in gene expression and gene-specific epigenetic modifications at the genome-wide level.

## Materials and methods

C2C12 (mouse myoblast) cell cultures were exposed to 1,000 µM WA for 24 h. ChIP assays were performed with cross-linked cells from WA-treated and untreated C2C12 cultures using anti-RNA Pol II, anti-phospho histone H3Ser10, anti-trimethyl histone H3K4 and anti-trimethyl histone H3K27 antibodies. ChIP assays were followed by sequencing where single-ended 50 bp reads were generated using a combination of the Applied Biosystems SOLiD™ and Illumina Hi-Seq™ systems. Data analysis was done using in-house developed pipelines (PERL/Unix) as well as several open source bioinformatics software from R/Bioconductor project and other sources.

## Results

Using different *in vitro* models, we examined metal-induced cytotoxicity and epigenetic modifications where WA showed cytotoxicity at concentrations >50 µg/ml, with C2C12 being relatively resistant to WA-mediated toxic impact. Using ChIP-Seq, we found several histone modifications up- and downregulated in the promoter regions of genes related to learning and memory mechanisms, with maximum impact observed for H3Ser10 phosphorylation. A total of 101 regions in the mouse genome were found to be most significantly depleted of H3S10 phosphorylation after WA treatment (fold change >3), these targets included several genes with neurological functions including voltage-dependent calcium channel (*CACNB1*), phosphatidylinositol 4-kinase type 2 (*Pi4k2a*) and Kinesin 5A (*KIF5A*)*.* In addition, pathway analysis of these 101 regions revealed 15 genes as part of regulatory networks that are responsible for developmental disorders, hereditary disorders and neurological diseases. ChIP-Seq analysis of other histone modifications and Pol II binding patterns are still ongoing. We plan to correlate expression levels investigated by RNA-Seq and epigenetic profiles impacted by WA exposure.

## Conclusions

Our results reveal epigenetic modifications triggered by WA exposure in C2C12 cells for the first time at a genome-wide level. In addition to epigenetic changes observed for specific genes, ChIP-Seq analysis confirmed our previous report on the gross genomic depletion of H3Ser10 phosphorylation [1]. Future investigations on genes identified in this study will help unravel the mechanisms involved in WA toxicity that may lead towards the development of therapeutics.

## References

[B1] Verma R Xu X Jaiswal MK Olsen C Mears D Caretti G Galdzicki Z In vitro profiling of epigenetic modifications underlying heavy metal toxicity of tungsten-alloy and its components Toxicol Appl Pharmacol 2011 253 178 187 10.1016/j.taap.2011.04.002 21513724

[B2] Pepke S Wold B Mortazavi A Computation for ChlP-seq and RNA-seq studies Nat Methods 2009 6 11 Suppl S22 32 19844228 10.1038/nmeth.1371 PMC4121056

